# *In silico* Analysis Excavates A Novel Competing Endogenous RNA Subnetwork in Adolescent Idiopathic Scoliosis

**DOI:** 10.3389/fmed.2020.583243

**Published:** 2020-10-28

**Authors:** Hui-Min Li, Yi Liu, Jing-Yu Ding, Renjie Zhang, Xiao-Ying Liu, Cai-Liang Shen

**Affiliations:** ^1^Department of Orthopedics & Spine Surgery, The First Affiliated Hospital of Anhui Medical University, Hefei, China; ^2^Department of Respiratory and Critical Care Medicine, The First Affiliated Hospital of Anhui Medical University, Hefei, China; ^3^School of Life Sciences, Anhui Medical University, Hefei, China

**Keywords:** adolescent idiopathic scoliosis, bioinformatics, Competing endogenous RNA (CeRNA), hub genes, lncRNAs, miRNAs

## Abstract

**Background and Objective:** Adolescent idiopathic scoliosis (AIS) is a complex three-dimensional deformity of the spine. Mesenchymal stem cells (MSCs) regulate bone mass homeostasis in AIS, which might be related to the pathogenesis of AIS. However, the mRNA–miRNA–lncRNA network linked to the regulation of the genetic pathogenesis of MSCs remains unknown.

**Methods:** We conducted an exhaustive literature search of PubMed, EMBASE, and the Gene Expression Omnibus database to find differentially expressed genes (DEGs), differentially expressed miRNAs (DE miRNAs), and differentially expressed lncRNAs (DE lncRNAs). Functional enrichment analysis was performed through Enrichr database. Protein–protein interaction (PPI) network was constructed using STRING database, and hub genes were identified by CytoHubba. Potential regulatory miRNAs and lncRNAs of mRNAs were predicted by miRTarBase and RNA22, respectively.

**Results:** We identified 551 upregulated and 476 downregulated genes, 42 upregulated and 12 downregulated miRNAs, and 345 upregulated and 313 downregulated lncRNAs as DEGs, DE miRNAs, and DE lncRNAs, respectively. Functional enrichment analysis revealed that they were significantly enriched in protein deglutamylation and regulation of endoplasmic reticulum unfolded protein response. According to node degree, one upregulated hub gene and eight downregulated hub genes were identified. After drawing the Venn diagrams and matching to Cytoscape, an mRNA–miRNA–lncRNA network linked to the pathogenesis of MSCs in AIS was constructed.

**Conclusion:** We established a novel triple regulatory network of mRNA–miRNA–lncRNA ceRNA, among which all RNAs may be utilized as the pathogenesis biomarker of MSCs in AIS.

## Introduction

Adolescent idiopathic scoliosis (AIS) is characterized by a complex 3D deformity of the spine, but its etiology and pathogenesis remain unclear (1–3). Abnormal skeletal growth and low bone mass are factors that may be associated with disturbed bone metabolism existing in patients with AIS (4–6). Recent studies have suggested that mesenchymal stem cells (MSCs) play a crucial role in bone mass homeostasis in AIS; however, the regulation of the genetic pathogenesis of MSCs remains yet to be elucidated (7, 8).

MicroRNAs (miRNAs) are a group of endogenous small single-stranded non-coding RNAs with a length of ~21–25 nucleotides (9). miRNAs can negatively regulate gene expression by binding to the 3′-untranslated region of messenger RNA (mRNA), leading to direct mRNA degradation or protein translation inhibition. Thus, miRNAs are involved in the regulation of many biological processes, such as proliferation, apoptosis, cell cycle, differentiation, and DNA repair (10). Competing endogenous RNA (ceRNA) hypothesis indicates that cross-talk between non-coding RNA (ncRNA) and mRNA is achieved by competitively binding to shared miRNAs (11, 12). Long non-coding RNAs (lncRNAs) are a class of ncRNA, which can decrease miRNA abundance as miRNA sponges and increase the expression of downstream target mRNAs of miRNAs (13, 14). Increasing evidence indicates that miRNAs and lncRNAs have an effect on the osteoblastic differentiation of MSCs (15–17). However, current knowledge of mRNA–miRNA–lncRNA in MSCs of AIS is limited. The aim of the present study is to explore and construct a key mRNA–miRNA–lncRNA ceRNA triple subnetwork in AIS based on comprehensive bioinformatics analysis.

In this study, we acquired differentially expressed genes (DEGs), differentially expressed miRNAs (DE miRNAs), and differentially expressed lncRNAs (DE lncRNAs) by mining previously published datasets and studies. The DEGs were analyzed by gene ontology (GO) annotation and Kyoto Encyclopedia of Genes and Genomes (KEGG) pathway enrichment analysis. Then, a protein–protein interaction (PPI) network was established to identify hub genes, and potential regulatory miRNAs were predicted. The key DE miRNAs were identified by drawing Venn diagrams, and the functions of the key DE miRNAs were also determined. Subsequently, potential regulatory lncRNAs were predicted, and the key DE lncRNAs were identified using Venn diagrams. A novel mRNA–miRNA–lncRNA regulatory network associated with the pathogenesis of patients with AIS was successfully established.

## Materials and Methods

### Search Literature and Microarray Assay

We conducted an exhaustive literature search of PubMed, EMBASE, and the Gene Expression Omnibus database (http://www.ncbi.nlm.nih.gov/geo/) for studies that compared the gene or miRNA or lncRNA expression in bone marrow MSCs from patients with AIS and those from healthy individuals. Studies were selected and excluded based on the following criteria: (1) The study population consisted of patients with AIS who underwent a comprehensive clinical and radiological examination to rule out other causes of scoliosis and to determine the diagnosis of AIS. (2) In the non-AIS patients' group, each of the subjects had a straight spine and normal forward bending test on physical examination, determined not to have any associated medical conditions or spinal deformity. (3) Bone marrow aspirates were obtained from both groups. (4) Studies containing more than five AIS samples and five normal samples were included, whereas studies based on animal models were excluded. The titles and abstracts of these studies were screened, and information on the studies of interest was further evaluated. The search terms included “adolescent idiopathic scoliosis,” “AIS,” “mesenchymal stem cells,” and “MSCs.” We did not limit the languages or publication date.

### Differential Expression Analysis

DEGs and DE miRNAs were obtained through literature search of previous studies (8, 18). Gene reannotation was performed, and “Limma” R package was used for differential expression analysis of lncRNAs. |FoldChange| > 1 and *p* < 0.05 were set as the cut-off criteria.

### Functional Analysis

The GO and KEGG pathways of DEGs were analyzed based on the Enrichr database (http://amp.pharm.mssm.edu/Enrichr/) by performing functional annotation (biological process, BP, molecular function, MF, and cellular component, CC) and pathway enrichment analysis. *p* < 0.05 was considered statistically significant (19, 20). Then, the top 10 enriched GO terms and KEGG pathways were downloaded from the webpage. The functions of key DE miRNAs were analyzed through TAM 2.0 tools (21, 22) by using the hypergeometric test to evaluate the overexpression of each miRNA set (257 sets) in the “miRNA list.”

### PPI Network

The potential PPI relationship of DEGs was mapped to the Search Tool for the Retrieval of Interacting Genes (STRING) database (http://string-db.org/), which is designed to analyze PPI information (23). PPI node pairs with a medium combined score ≥0.4 were selected for further analysis. The PPI network was then visualized using the Cytoscape software (version 3.7.2, www.cytoscape.org/). Nodes with a high degree of connectivity are essential in maintaining the stability of the entire network (24). CytoHubba is a plugin in Cytoscape that calculates the degree of each protein node. In our study, the top 30 hub genes were visualized in Cytoscape, and the top 10 hub genes were chosen for subsequent analysis.

### Prediction and Validation of miRNA and Construction of the mRNA–miRNA Network

The miRTarbase database, which collects experimentally validated microRNA-target interactions by a reporter assay, Western blot analysis, qPCR, microarray, and next-generation sequencing experiments, was used to predict the potential miRNAs binding to hub genes (25). Then, the common DE miRNAs between predicted and previous DE miRNAs were identified as the key DE miRNAs by drawing Venn diagrams. The network of key genes and miRNA was constructed through the Cytoscape software.

### Prediction and Validation of lncRNA and Construction of the mRNA–miRNA–lncRNA Network

The RNA22 tool was used to predict the potential lncRNAs binding to previously identified commonly DE miRNAs (26). Then, the commonly DE lncRNAs between predicted and previously DE lncRNAs were identified as the key DE lncRNAs by drawing Venn diagrams. The ceRNA network was constructed through the Cytoscape software.

## Results

### Study Search and Identification of DEGs, DE miRNAs, and DE lncRNAs

**Figure 1:**
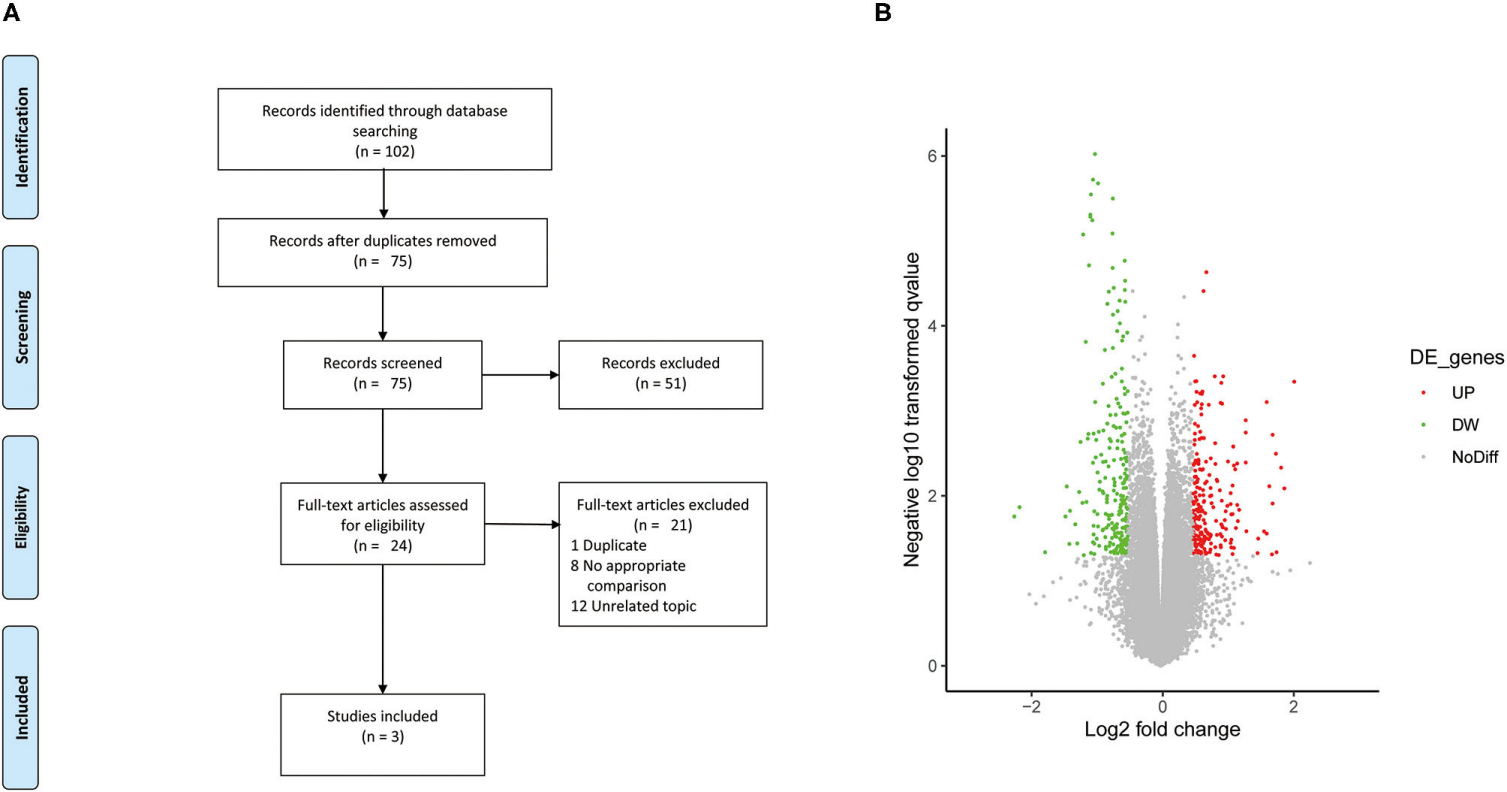
**(A)** Flow diagram of study selection. **(B)** Volcano plot of the DE lncRNAs. The black dots represent lncRNAs that are not differentially expressed, and the red dots and green dots represent the upregulated and downregulated lncRNAs, respectively.

A summary of the study selection process is shown in Figure 1A. A total of 102 relevant studies were inspected via electronic search. A total of 27 duplicate studies were excluded. After the titles and abstracts were assessed, 51 studies that did not meet the eligibility criteria were eliminated. After the full text of the remaining 24 studies was verified, only three studies were included (8, 17, 18). The gene dataset was performed on the Affymetrix Gene Chip Human Transcript 2.0 arrays containing ten human AIS samples and five non-AIS samples and as did the miRNA dataset. The lncRNA dataset GSE110359 was also analyzed on the Affymetrix Gene Chip Human Transcript 2.0 arrays containing 12 human AIS samples and five non-AIS samples. Gene, miRNA, lncRNA datasets of the AIS group and the control group were from three different cohorts.

A total of 1,027 genes (551 upregulated and 476 downregulated genes) and 54 miRNAs (42 upregulated and 12 downregulated miRNAs) were extracted from the included studies (8, 18) and identified as DEGs and DE miRNAs. Based on differential expression analysis, 658 lncRNAs (345 upregulated and 313 downregulated lncRNAs) were identified as DE lncRNAs, and the volcano plots of these DE lncRNAs is shown in Figure 1B.

### GO and KEGG Analyses of DEGs

**Figure 2:**
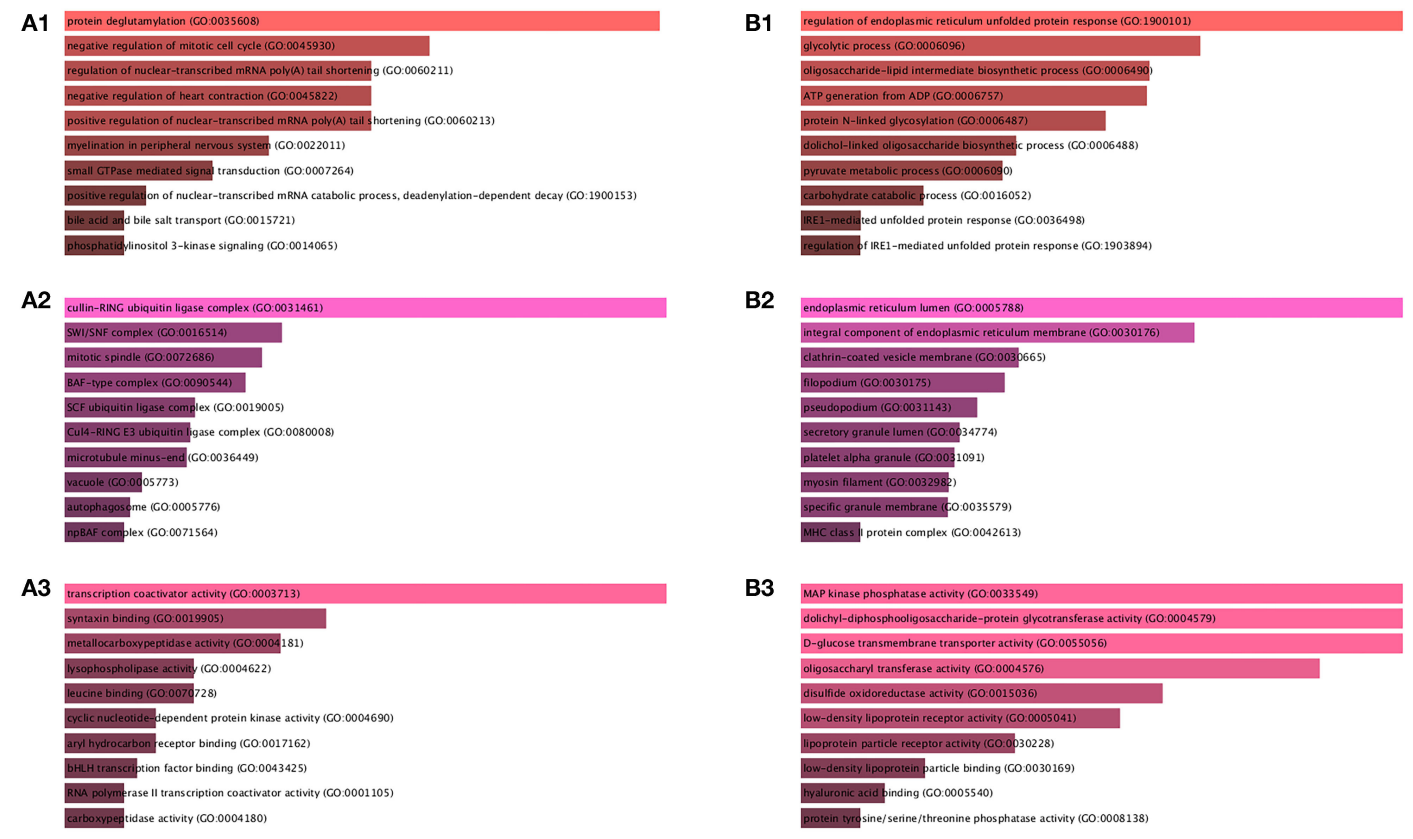
GO functional annotation for the significant DEGs. **(A1)** The top 10 enriched BP of the upregulated significant DEGs. **(A2)** The top 10 enriched CC of the upregulated significant DEGs. **(A3)** The top 10 enriched MF of the upregulated significant DEGs. **(B1)** The top 10 enriched BP of the downregulated significant DEGs. **(B2)** The top 10 enriched CC of the downregulated significant DEGs. **(B3)** The top 10 enriched MF of the downregulated significant DEGs. Ranking by combined score. Red color indicates *p* < 0.05 in BP; Pink color indicates *p* < 0.05 in CC; Jacinth color indicates *p* < 0.05 in MF.

**Figure 3:**
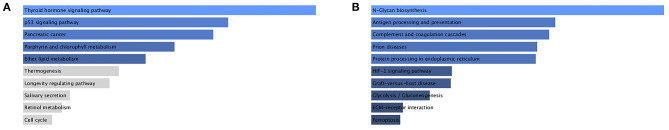
KEGG pathway enrichment analysis for the significant DEGs. **(A)** Upregulated significant DEGs. **(B)** Downregulated significant DEGs. Ranking by combined score. Blue color indicates *p* < 0.05; Gray color indicates *p* ≥ 0.05.

As shown in Figures 2A1–A3, the upregulated genes between AIS and normal samples are significantly enriched in protein deglutamylation, negative regulation of mitotic cell cycle, and positive regulation of nuclear-transcribed mRNA poly (A) tail shortening in the BP category, cullin-RING ubiquitin ligase complex, SWI/SNF complex, and mitotic spindle in the CC category and in transcription coactivator activity, syntaxin binding, and metallocarboxypeptidase activity in the MF category. KEGG enrichment analysis for these DEGs revealed that the thyroid hormone signaling pathway, p53 signaling pathway, and pancreatic cancer are significantly enriched pathways (Figure 3A).

As shown in Figures 2B1–B3, downregulated genes between AIS and normal samples were significantly enriched in regulation of endoplasmic reticulum unfolded protein response, glycolytic process, and oligosaccharide-lipid intermediate biosynthetic process in the BP category; endoplasmic reticulum lumen, integral component of endoplasmic reticulum membrane, and clathrin-coated vesicle membrane in the CC category; and MAP kinase phosphatase activity, dolichyl-diphosphooligosaccharide-protein glycotransferase activity, and D-glucose transmembrane transporter activity in the MF category. KEGG enrichment analysis for these DEGs revealed that N-glycan biosynthesis, antigen processing and presentation, and complement and coagulation cascades were significantly enriched pathways (Figure 3B).

### PPI Network Construction and Hub Gene Identification

**Figure 4:**
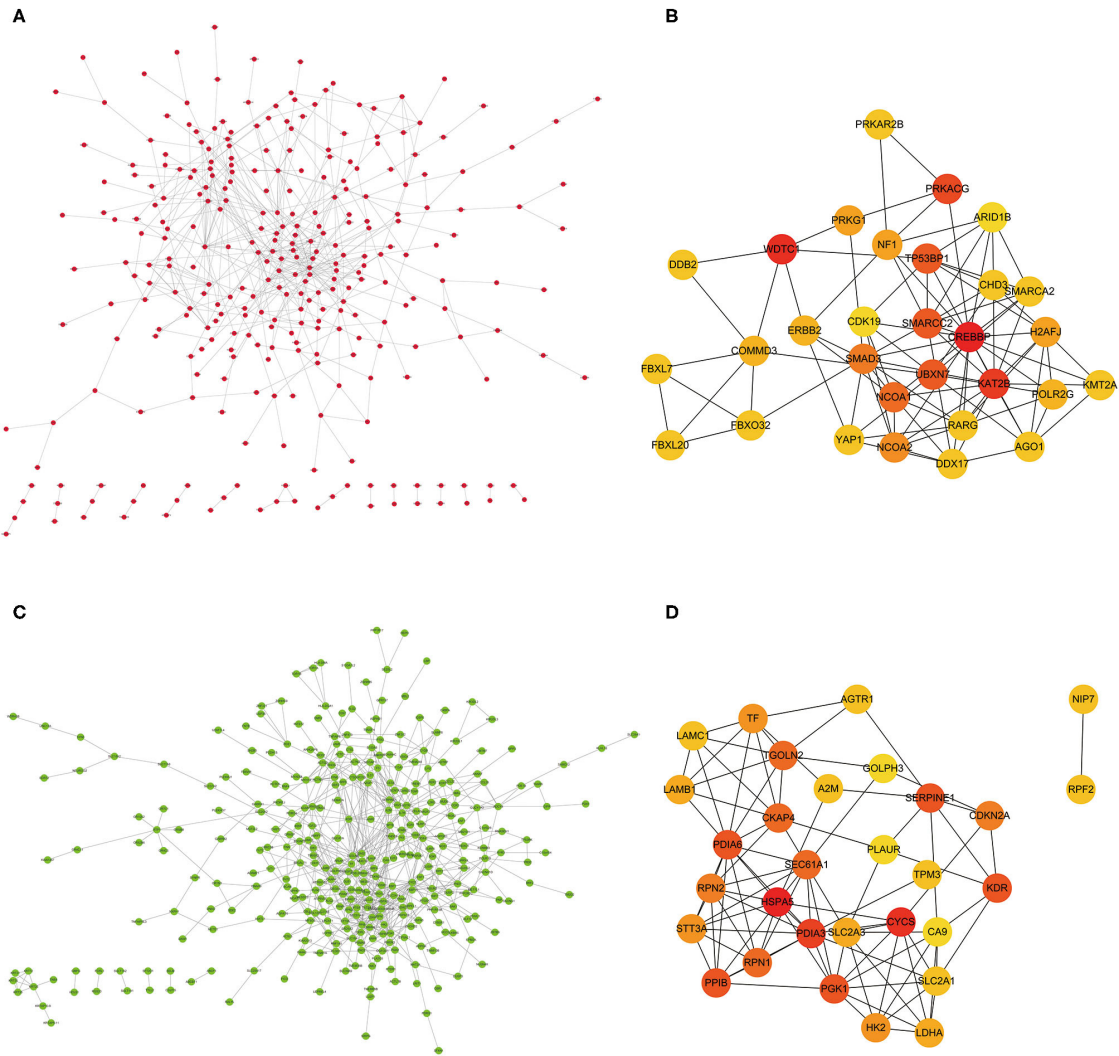
**(A)** PPI network of the significant upregulated DEGs. **(B)** The top 30 hub genes of the significant upregulated DEGs. **(C)** The PPI network of the significant downregulated DEGs. **(D)** The top 30 hub genes of the significant downregulated DEGs.

Protein interactions among the DEGs were predicted with STRING tools. A total of 403 nodes and 537 edges were involved in the PPI network (PPI enrichment *p* < 7.47e−08) of upregulated DEGs, as shown in Figure 4A. The top 30 upregulated genes are shown in Figure 4B and Table 1. A total of 437 nodes and 739 edges were involved in the PPI network (PPI enrichment *p* < 1.0e−16) of downregulated DEGs, as presented in Figure 4C, and the top 30 downregulated genes are shown in Figure 4D and Table 1. The top 10 upregulated hub genes included *CREBBP, WDTC1, KAT2B, PRKACG, TP53BP1, UBXN7, SMARCC2, NCOA1, SMAD3*, and *NCOA2*. The top ten downregulated hub genes included *HSPA5, CYCS, PDIA3, KDR, PGK1, PDIA6, SERPINE1, PPIB, CKAP4*, and *TGOLN2*. Those 20 hub genes were chosen for the following analyses.

**Table 1:** The top 30 hub genes in PPI networks.

**Upregulated gene**	Degree*	**Downregulated gene**	Degree*
CREBBP	33	HSPA5	31
WDTC1	23	CYCS	27
KAT2B	20	PDIA3	19
PRKACG	18	KDR	16
TP53BP1	16	PGK1	16
UBXN7	16	PDIA6	16
SMARCC2	16	SERPINE1	16
NCOA1	15	PPIB	16
SMAD3	13	CKAP4	15
NCOA2	12	TGOLN2	15
NF1	11	SEC61A1	15
PRKG1	11	RPN1	15
H2AFJ	11	CDKN2A	14
COMMD3	10	RPN2	14
POLR2G	10	HK2	13
ERBB2	10	TF	13
FBXL7	9	STT3A	13
FBXL20	9	LDHA	12
FBXO32	9	SLC2A3	12
YAP1	9	LAMB1	12
DDX17	9	SLC2A1	11
KMT2A	9	AGTR1	11
RARG	9	A2M	11
AGO1	9	TPM3	11
DDB2	9	NIP7	11
CHD3	9	RPF2	11
PRKAR2B	9	LAMC1	11
SMARCA2	9	GOLPH3	10
HECW2	8	CA9	10
CUL4B	8	FGF23	10

* *Degree indicates connectivity*.

### Prediction and Validation of Potential Key miRNAs of Key Genes

**Figure 5:**
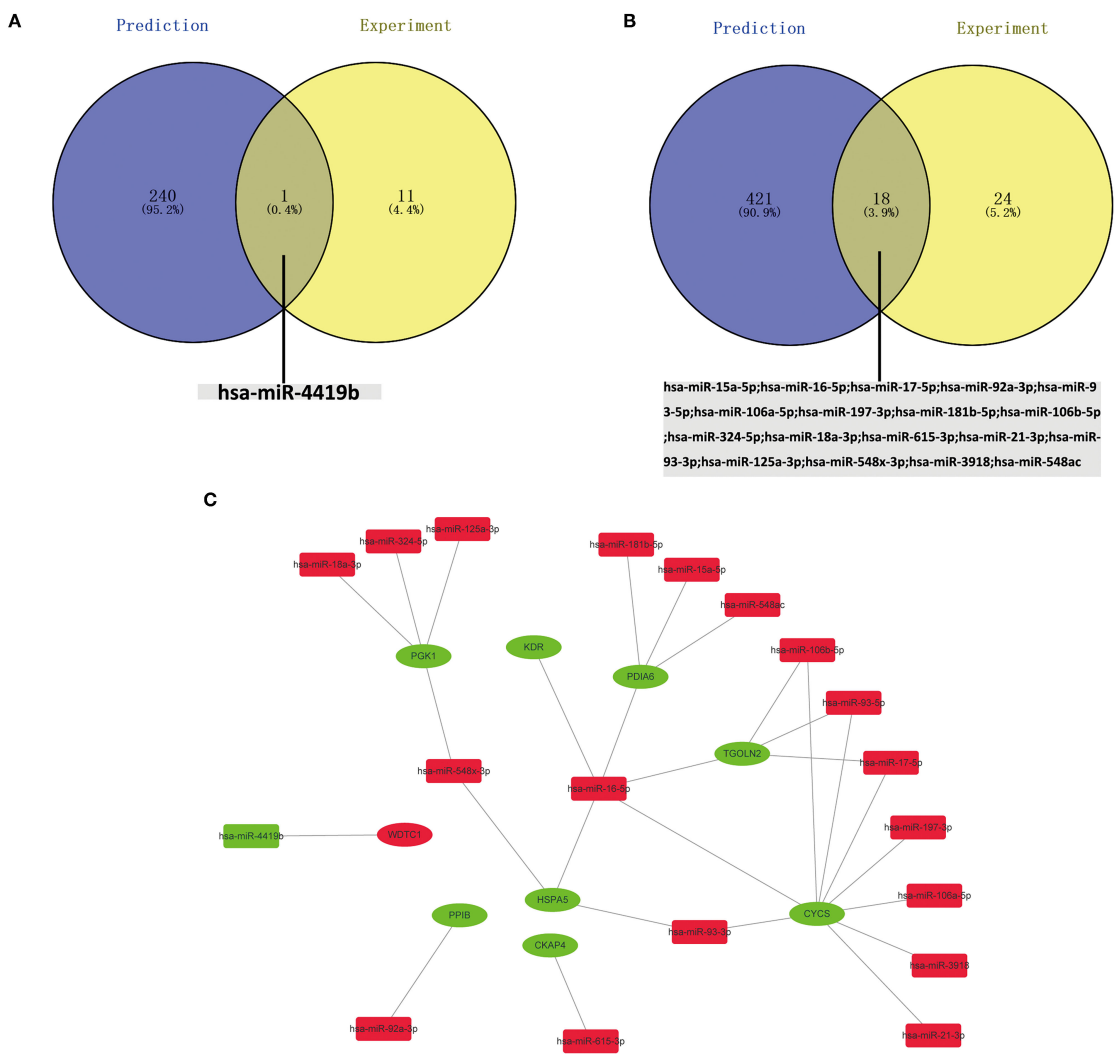
**(A)** Identification of key downregulated miRNAs by combining prediction and experiment analyses. **(B)** Identification of key unregulated miRNAs by combining prediction and experiment analyses. **(C)** Construction of the miRNA–gene network.

The potential miRNAs binding to the 20 key genes were predicted through the miRTarBase, and a total of 241 miRNAs were identified for upregulated DEGs. After Venn diagrams were constructed with DE miRNAs, one downregulated miRNA that could potentially regulate one key gene (*WDTC1*) expression was identified as presented in Figure 5A. Potential miRNAs binding to the nine key upregulated genes (*CREBBP, KAT2B, PRKACG, TP53BP1, UBXN7, SMARCC2, NCOA1, SMAD3*, and *NCOA2*) were not observed. For downregulated hub genes, a total of 439 miRNAs were identified. After the construction of Venn diagrams with DE miRNAs, we identified 18 upregulated miRNAs that could potentially regulate eight key gene (*HSPA5, CYCS, KDR, PGK1, PDIA6, PPIB, CKAP4*, and *TGOLN2*) expression as presented in Figure 5B. Potential miRNAs binding to two key downregulated genes (*PDIA3* and *SERPINE1*) were not observed.

### Construction of the Key mRNA–miRNA Network in AIS

Based on the classical inverse relationship between miRNA and target gene, the 18 potential upregulated miRNAs binding to eight downregulated genes (*HSPA5, CYCS, KDR, PGK1, PDIA6, PPIB, CKAP4*, and *TGOLN2*) were identified, and only one potential downregulated miRNA binding to an upregulated gene (*WDTC1*) was identified. The network (*miR-93-3p/miR-16-5p/miR-548x-3p - HSPA5, miR-16-5p/miR-17-5p/miR-93-5p/miR-106a-5p/miR-197-3p/miR-106b-5p/miR-21-3p/miR-93-3p/miR-3918 - CYCS, miR-16-5p - KDR, miR-324-5p/miR-18a-3p/miR-125a-3p/miR-548x-3p - PGK1, miR-15a-5p/miR-16-5p/miR-181b-5p/miR-548ac - PDIA6, miR-92a-3p - PPIB, miR-615-3p - CKAP4, miR-16-5p/miR-17-5p/miR-93-5p/miR-106b-5p - TGOLN2, miR-4419b - WDTC1*) of key mRNA and miRNA is shown in Figure 5C.

### Functional Analysis of Key miRNAs

**Figure 6:**
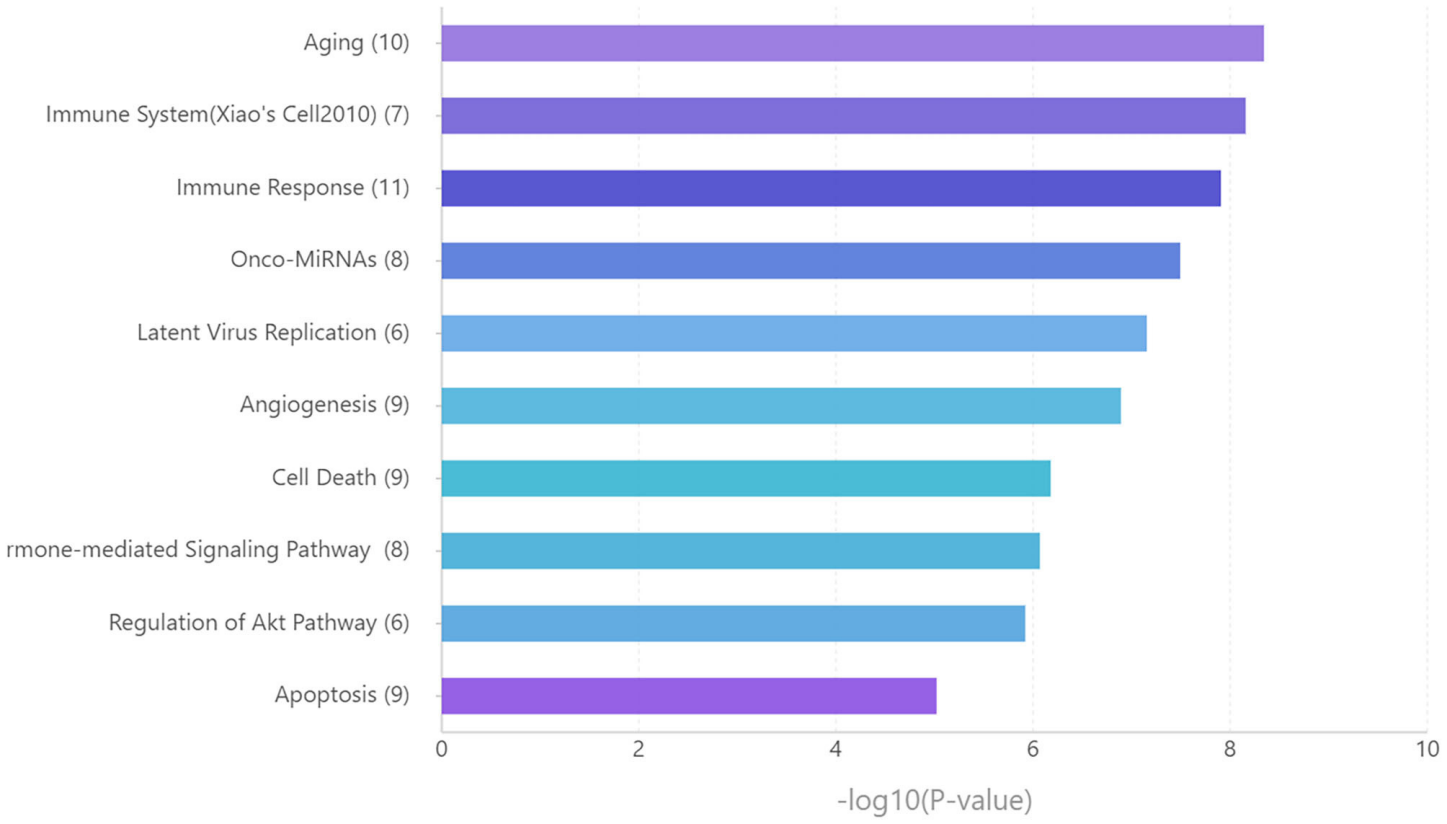
Functional enrichment analysis for the key miRNAs. Ranking by –log 10 (*p*-value).

Functional enrichment analysis for these miRNAs revealed that aging, immune system, and immune response are significantly enriched pathways (Figure 6).

### Prediction and Validation of Potential Key lncRNAs Binding to Key miRNAs

**Figure 7:**
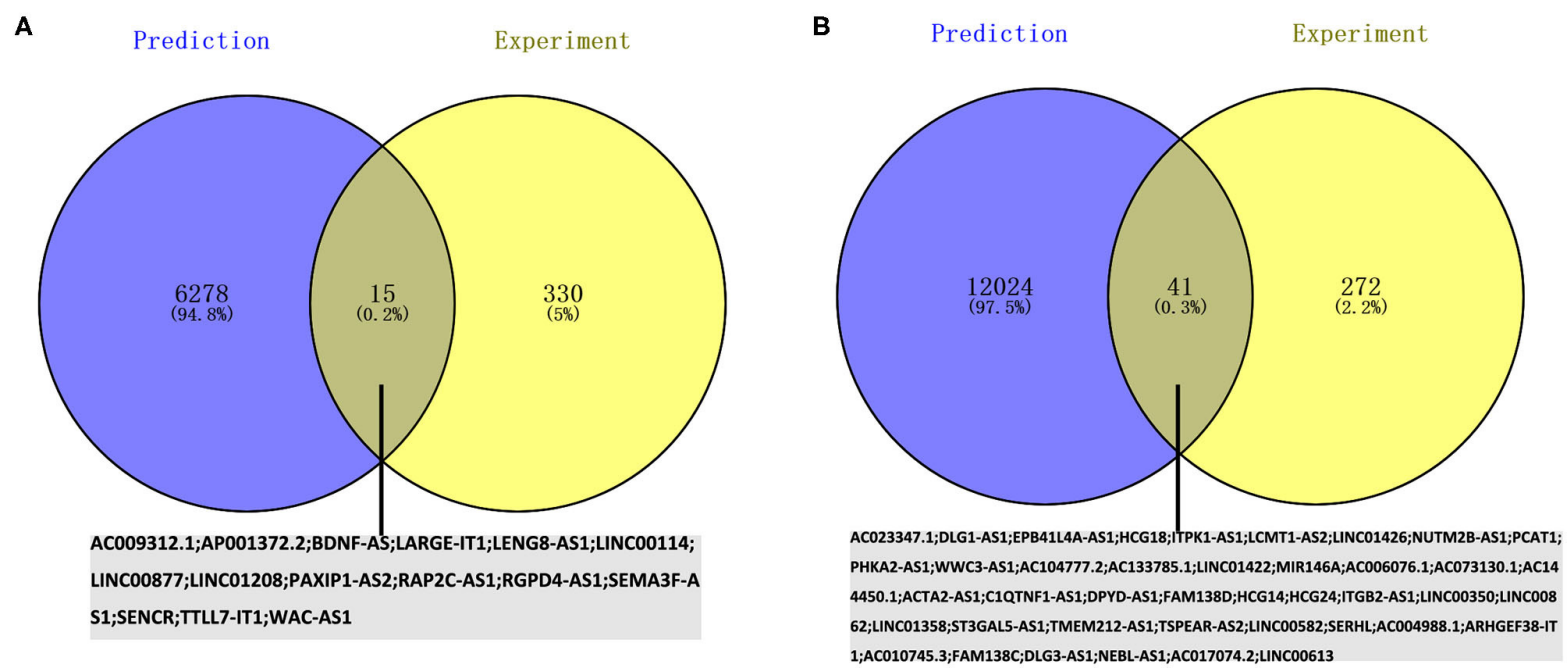
Screening the key lncRNAs in AIS. **(A)** Identification of key upregulated miRNAs by combining prediction and experiment analyses. **(B)** Identification of key downregulated miRNAs by combining prediction and experiment analyses.

Given that no direct database can accommodate queries on the pairing relationship between lncRNAs and miRNAs in large quantities, RNA22 was used to predict the potential lncRNAs binding to the 19 key miRNAs, and a total of 6,293 lncRNAs were identified for downregulated miRNAs. After Venn diagrams with previous DE lncRNAs were constructed, 15 lncRNAs that could potentially regulate one key downregulated miRNA (*miR-4419b*) expression was identified as presented in Figure 7A. A total of 12,065 lncRNAs were identified for upregulated miRNAs. After Venn diagrams with previous DE lncRNAs were constructed, 41 lncRNAs that could potentially regulate the expression of 17 key miRNAs (*miR-15a-5p, miR-16-5p, miR-17-5p, miR-92a-3p, miR-93-5p, miR-106a-5p, miR-197-3p, miR-181b-5p, miR-106b-5p, miR-324-5p, miR-18a-3p, miR-615-3p, miR-21-3p, miR-93-3p, miR-125a-3p, miR-3918*, and *miR-548x-3p*) were identified as shown in Figure 7B. Potential lncRNAs binding to one key upregulated miRNA (*miR-548ac*) were not observed.

### Construction of Key mRNA–miRNA–lncRNA Triple Subnetwork in AIS

**Figure 8:**
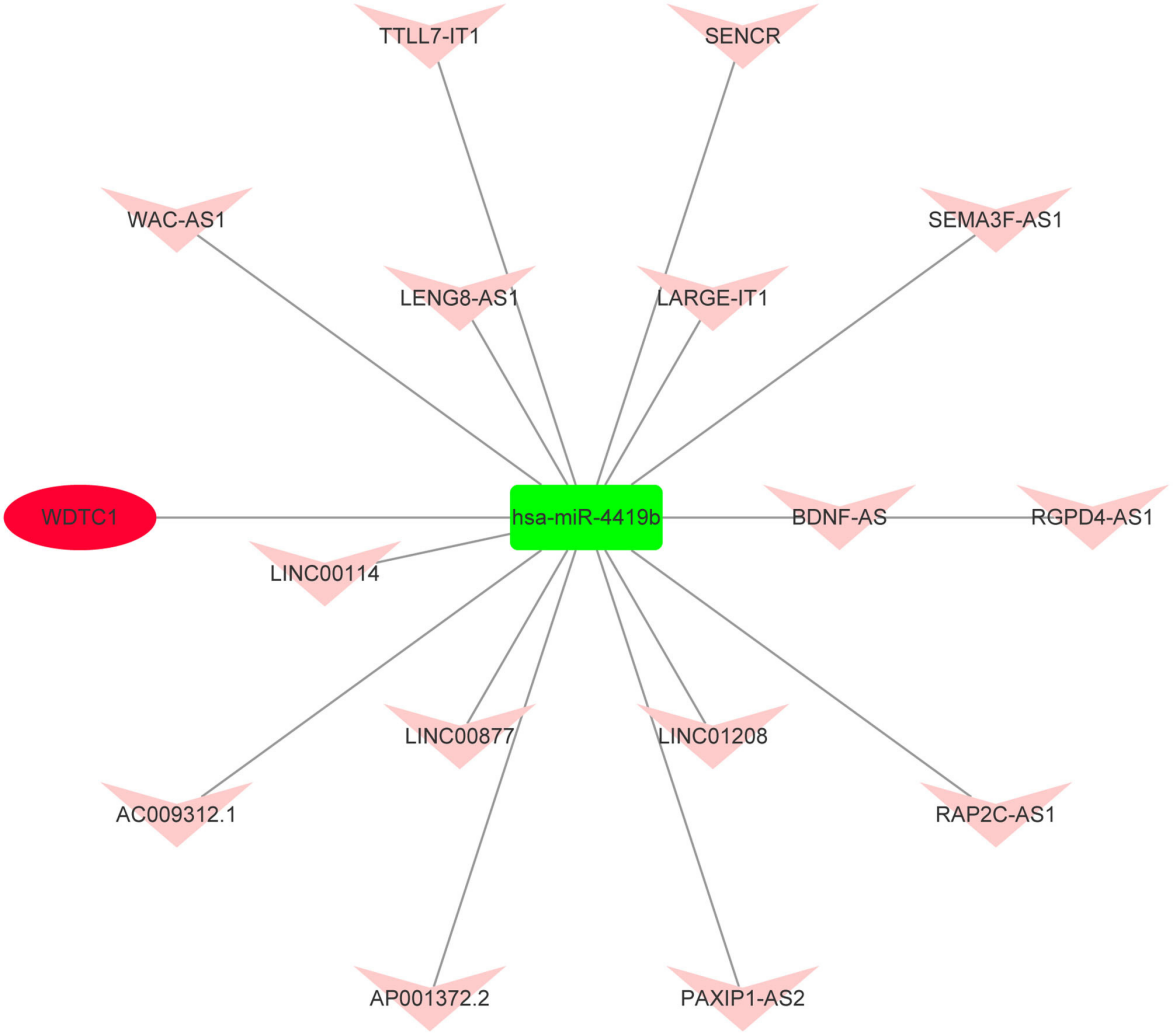
Novel upregulated key mRNA's competing endogenous RNA (ceRNA) triple regulatory network. The green rectangle in the network represents downregulated miRNAs. The red ellipse in the network represents upregulated hub genes. The red V in the network represents upregulated lncRNAs.

**Figure 9:**
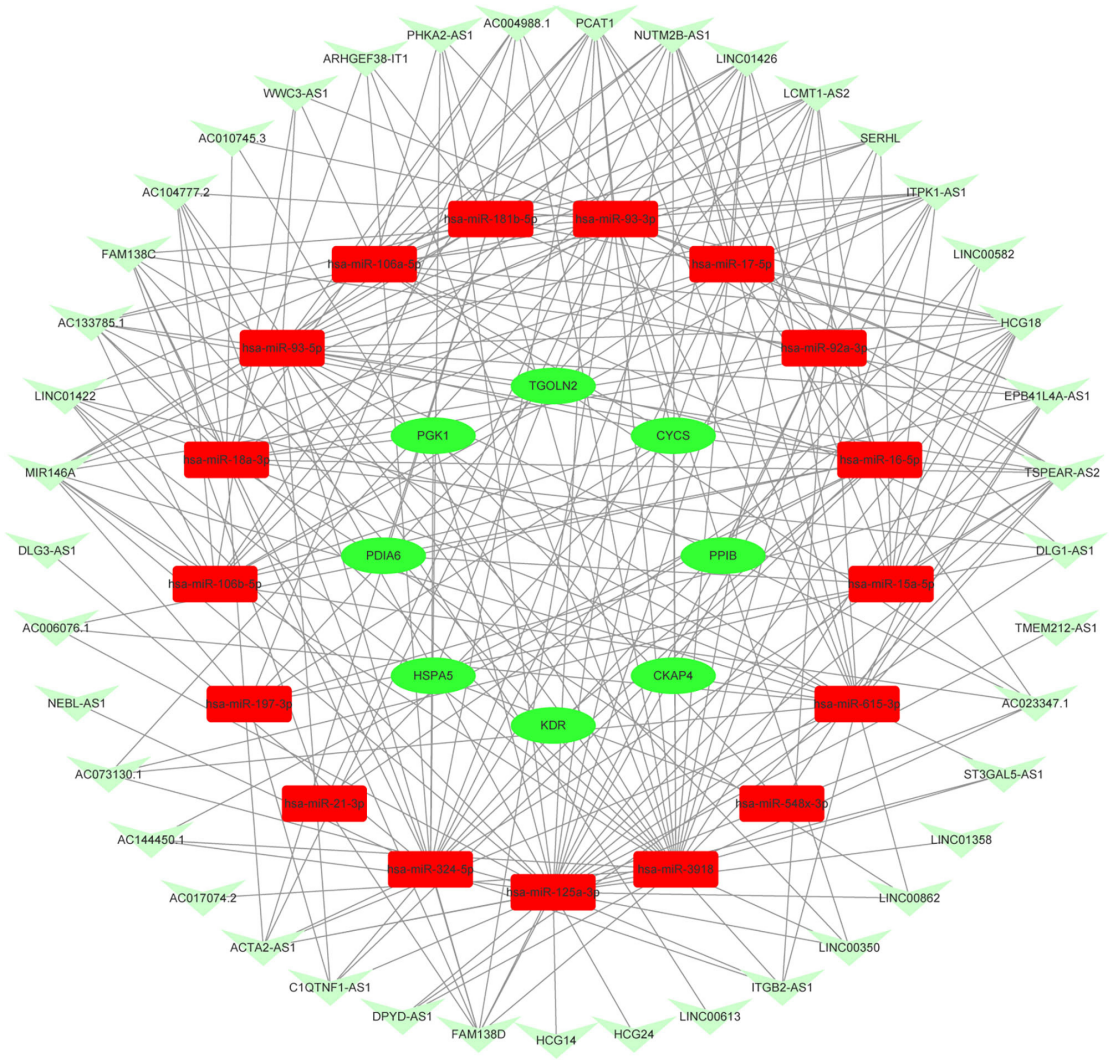
Novel downregulated key mRNAs' ceRNA triple regulatory network. The red rectangle in the network represents upregulated miRNAs. The green ellipse in the network represents downregulated hub genes. The green V in the network represents downregulated lncRNAs.

**Figure 10:**
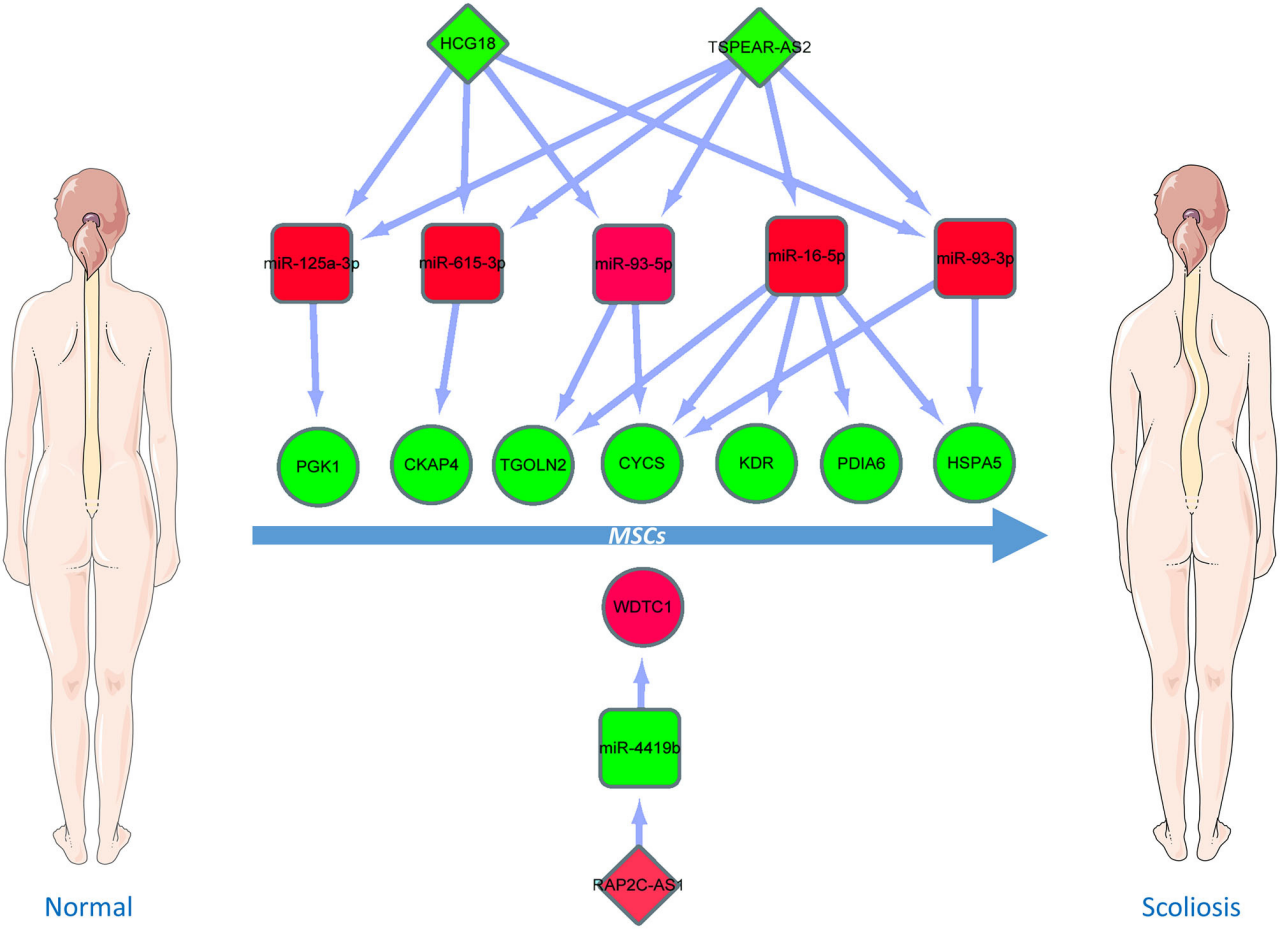
Critical ceRNA triple regulatory network. The rectangle in the network represents miRNAs. The ellipse in the network represents hub genes. The diamond in the network represents lncRNAs. Green represents downregulation, while red represents upregulation.

Based on the aforementioned analysis, an mRNA–miRNA–lncRNA network was constructed using Cytoscape. The triple subnetwork of upregulated and downregulated hub genes is shown in Figures 8, 9. The detailed information of subnetworks is listed in Supplementary Tables 1, 2. Each of the 18 miRNAs (*miR-15a-5p, miR-16-5p, miR-17-5p, miR-92a-3p, miR-93-5p, miR-106a-5p, miR-197-3p, miR-181b-5p, miR-106b-5p, miR-324-5p, miR-18a-3p, miR-615-3p, miR-21-3p, miR-93-3p, miR-125a-3p, miR-3918, miR-548x-3p*, and *miR-4419b*) was regulated by lncRNAs, which then regulated the target genes of miRNAs. The differential expression level of *lncRNA RAP2C-AS1* was the highest in the network regulating *miR-4419b*, and other certain lncRNAs (*HCG18* and *TSPEAR-AS2*) and miRNAs (*miR-16-5p, miR-93-3p*, and *miR-93-5p*) may play important roles in the ceRNA network as they regulate the majority of the downregulated hub genes. Therefore, the *RAP2C-AS1 - miR-4419b - WDTC1, TSPEAR-AS2 - miR-16-5p - CYCS/KDR/PDIA6/TGOLN2/HSPA5, TSPEAR-AS2/HCG18 - miR-93-3p - CYCS/HSPA5, TSPEAR-AS2/HCG18 - miR-93-5p - CYCS/ TGOLN2, TSPEAR-AS2/HCG18 - miR-615-3p - CKAP4*, and *TSPEAR-AS2/HCG18 - miR-125a-3p - PGK1* axes may play critical roles in the network (Figure 10).

## Discussion

This study was designed to investigate the specific ceRNA network based on the “mRNA–miRNA–lncRNA” order pattern. A total of 1,027 significant DEGs, consisting of 551 upregulated and 476 downregulated DEGs were extracted from the included studies. These genes are involved in multiple biological processes, including protein deglutamylation, small GTPase-mediated signal transduction, phosphatidylinositol 3-kinase signaling, RNA polymerase II transcription coactivator activity, MAP kinase phosphatase activity, negative regulation of the mitotic cell cycle, regulation of endoplasmic reticulum-unfolded protein response, glycolytic process, and positive regulation of the nuclear-transcribed mRNA poly (A) tail shortening. Although Zhuang et al. (8) used a different functional annotation database, they found that small GTPase-mediated signal transduction, phosphatidylinositol 3-kinase signaling, RNA polymerase II transcription coactivator activity, and MAP kinase phosphatase activity were enriched in GO analysis of DEGs. Elevated levels of the small GTPase Cdc42 could induce senescence in male rat MSCs (27). Domingues et al. (28) found that Cofilin-1 levels were elevated in human mesenchymal stem/stromal cells cultured on soft substrates, which could promote Cofilin-1-dependent increased RNA transcription and faster RNA polymerase II-mediated transcription elongation. It has been reported that *miR-16* regulated MgCl2-induced promotion of osteogenic differentiation of MSCs by targeting *FGF2*-mediated activation of the *ERK/MAPK* pathway (29). Meanwhile, the pathway enrichment analysis of DEGs and key miRNAs revealed the presence of multiple enriched pathways, including the thyroid hormone signaling pathway, p53 signaling pathway, aging, regulation of *Akt* pathway, and apoptosis. Yoon et al. (30) have found that NRF2 could determine the self-renewal and osteogenic differentiation potential of human MSCs via the P53-SIRT1 axis. Other teams reported that *PI3K/Akt* signaling is the main contributor to MSC proliferation in response to *PDGFR*β activation and *Erk* activation by *PDGFR*β signaling, potently inhibiting the adipocytic differentiation of MSCs by blocking PPARγ and CEBPα expression (31). Through the activation of the AKT/glycogen synthase kinase signaling pathway and suppression of apoptosis, *PBX* homeobox 1 enhances hair follicle MSC proliferation and reprogramming. Thus, these significant DEGs may be involved in the molecular pathogenesis of AIS initiation and development.

To analyze the relationships and functions of significant DEGs, we obtained PPI networks from STRING database and identified one upregulated hub gene (*WDTC1*) and eight downregulated hub genes (*HSPA5, CYCS, KDR, PGK1, PDIA6, PPIB, CKAP4*, and *TGOLN2*) that may be related to the pathogenesis of AIS. Most of these key genes have been well-investigated in MSCs. For example, *WDTC1* is a conserved and single-copy gene in humans (32) and studies have linked the function of *WDTC1* to the negative regulation of adipogenesis (33, 34). Besides, Liang et al. have reported that the adipogenic ability of MSCs from AIS girls was lower than that of controls (35). *HSPA5* encodes heat shock protein 70 (HSP70), which was involved in proliferation, differentiation, and apoptosis of MSCs (36, 37). *CYCS* positively regulates MSC apoptosis (16) and is involved in neuronal developmental disorders, amyotrophic lateral sclerosis, and neuron death (38). *KDR*, also known as VEGF receptor 2, is a critical regulator of MSC osteogenesis following the activation of the *ERK/RUNX2* signaling pathway (39). *PGK1* overexpression may induce osteoblastic differentiation of bone marrow stromal cells and inhibit osteoclastogenesis (40). These publications partially support the accuracy of potential core genes found in our bioinformatics analyses, while the function of these core genes in MSCs of AIS requires further research.

MiRNAs and lncRNAs are involved in regulating gene expression and function through the mechanism of ceRNA. Previous studies have shown that some miRNAs binding to the key genes that we screened were identical to our analytic results. For example, *miR-17-5p* and *miR-106a* upregulation could promote adipogenesis and inhibit osteogenesis by targeting BMP2 in the modulation of human adipose-derived MSCs (41). By targeting *Smad5, miR-17-5p* and *miR-106b-5p* could suppress the osteogenic differentiation of C2C12 cells and inhibit bone formation (42). *miR-16-5p* inhibits osteoclastogenesis in giant cell tumor of bone via the direct inhibition of receptor activator of nuclear factor-κB ligand- (RANKL-) (15). Downregulation of *miRNA-16-5p* could accelerate fracture healing by negatively regulating *Bcl-2* and *Cyclin-D1* expression in MC3T3-E1 cells (43). *miR-93-5p* suppresses the osteogenic differentiation of mouse MSCs (C3H10T1/2) by targeting *Smad5* (44). In trauma-induced osteonecrosis of patients with femoral head fractures, increased *miRNA-93-5p* inhibits osteogenic differentiation by targeting bone morphogenetic protein-2 (45). Considering that the ceRNA hypothesis indicates that cross-talk between lncRNAs and mRNAs is achieved through competitive binding to shared miRNAs, 56 potential key lncRNAs binding to these key miRNAs were identified. However, studies on the role of lncRNA of AIS are few. Only the group of Zhuang has found a novel *lncAIS* (gene symbol: ENST00000453347) that could suppress osteogenic differentiation of MSCs in AIS (17). Upregulation of *BDNF-AS* promotes bone marrow-derived MSCs' self-proliferation but inhibits osteogenic differentiation through *BDNF* regulation (46); *BDNF-AS* expression was also upregulated in this study. A pathogenesis-associated mRNA–miRNA–lncRNA network in AIS was successfully established, and such a network has been identified in previous literature on osteogenic differentiation. For example, downregulated FAM83H-AS1 modulates the SpA-inhibited osteogenic differentiation in human bone MSCs by *FAM83H-AS1/miR-541-3p/WNT3A* axis (47). *lncRNA MSC-AS1* could promote osteogenic differentiation of bone marrow-derived MSCs through sponging *miRNA-140-5p* to upregulate *BMP2* (48). Collectively, although a series of bioinformatics analyses has yielded some intriguing findings in our current study, further laboratory experiments and large-scale clinical trials are needed in the future. Besides, the artificial selection of ten upregulated and downregulated genes may result in some risk of selection bias.

## Conclusion

In summary, through integrated bioinformatics analysis and exhaustive literature search, we constructed a novel triple regulatory network of mRNA–miRNA-lncRNA ceRNA, in which all RNAs have significant predictive value for the pathogenesis of MSCs in AIS. We also identified that the *RAP2C-AS1 - miR-4419b - WDTC1, TSPEAR-AS2 - miR-16-5p - CYCS/KDR/PDIA6/TGOLN2/HSPA5, TSPEAR-AS2/HCG18 - miR-93-3p - CYCS/HSPA5, TSPEAR-AS2/HCG18 - miR-93-5p - CYCS/ TGOLN2, TSPEAR-AS2/HCG18 - miR-615-3p – CKAP4* and *TSPEAR-AS2/HCG18 - miR-125a-3p - PGK1* axes may play critical roles in the ceRNA network. In addition to the prognostic value of this mRNA–miRNA–lncRNA network for AIS, our findings provide important insights into the molecular mechanism of AIS. However, further studies are needed to validate these findings.

## Data Availability Statement

Publicly available datasets were analyzed in this study. This data can be found here: lncRNA (GSE110359); gene and miRNA datasets come from the Supplementary Material in (8, 18).

## Author Contributions

C-LS and X-YL designed the study. H-ML wrote the manuscript. YL and J-YD revised and polished the manuscript. H-ML, YL, J-YD, and RZ performed the statistical analysis of the data. All authors read and approved the final manuscript.

## Conflict of Interest

The authors declare that the research was conducted in the absence of any commercial or financial relationships that could be construed as a potential conflict of interest.

## Abbreviations

AIS: Adolescent idiopathic scoliosis
MSCs: mesenchymal stem cells
miRNA: microRNA
lncRNA: long non-coding RNA
ceRNA: competing endogenous RNA
GEO: Gene Expression Omnibus
DEGs: differentially expressed genes
GO: gene ontology
KEGG: Kyoto Encyclopedia of Genes and Genomes
PPI: protein-protein interaction
BP: biological process
MF: molecular function
CC: cellular component
STRING: Search Tool for the Retrieval of Interacting Genes.
