# Supplementary material for: In silico Analysis Excavates A Novel Competing Endogenous RNA Subnetwork in Adolescent Idiopathic Scoliosis
Source: Front Med (Lausanne). 2020 Oct 28;7:583243. doi: 10.3389/fmed.2020.583243 (PMC7655901; doi:10.3389/fmed.2020.583243)
Supplement: Supplementary file 1 [file Table_1.DOCX]

Table S1.The novel upregulated key mRNA’s competing endogenous RNA (ceRNA) triple regulatory network

| Upregulated LncRNA | Downregulated miRNA | Upregulated gene |
| --- | --- | --- |
| AC009312.1 | hsa-miR-4419b | WDTC1 |
| AP001372.2 | hsa-miR-4419b | WDTC1 |
| BDNF-AS | hsa-miR-4419b | WDTC1 |
| LARGE-IT1 | hsa-miR-4419b | WDTC1 |
| LENG8-AS1 | hsa-miR-4419b | WDTC1 |
| LINC00114 | hsa-miR-4419b | WDTC1 |
| LINC00877 | hsa-miR-4419b | WDTC1 |
| LINC01208 | hsa-miR-4419b | WDTC1 |
| PAXIP1-AS2 | hsa-miR-4419b | WDTC1 |
| RAP2C-AS1 | hsa-miR-4419b | WDTC1 |
| RGPD4-AS1 | hsa-miR-4419b | WDTC1 |
| SEMA3F-AS1 | hsa-miR-4419b | WDTC1 |
| SENCR | hsa-miR-4419b | WDTC1 |
| TTLL7-IT1 | hsa-miR-4419b | WDTC1 |
| WAC-AS1 | hsa-miR-4419b | WDTC1 |
