# Supplementary material for: In silico Analysis Excavates A Novel Competing Endogenous RNA Subnetwork in Adolescent Idiopathic Scoliosis
Source: Front Med (Lausanne). 2020 Oct 28;7:583243. doi: 10.3389/fmed.2020.583243 (PMC7655901; doi:10.3389/fmed.2020.583243)
Supplement: Supplementary file 2 [file Table_2.DOCX]

Table S2. The novel downregulated key mRNAs’ competing endogenous RNA (ceRNA) triple regulatory network.

| Downregulated lncRNA | Upwnregulated miRNA | Downregulated gene |
| --- | --- | --- |
| AC023347.1 |  |  |
| DLG1-AS1 |  |  |
| EPB41L4A-AS1 |  |  |
| HCG18 |  |  |
| ITPK1-AS1 |  |  |
| LCMT1-AS2 | hsa-miR-106a-5p | CYCS |
| LINC01426 |  |  |
| NUTM2B-AS1 |  |  |
| PCAT1 |  |  |
| PHKA2-AS1 |  |  |
| WWC3-AS1 |  |  |
| AC023347.1 |  |  |
| EPB41L4A-AS1 |  |  |
| HCG18 |  |  |
| ITPK1-AS1 |  |  |
| LINC01426 |  |  |
| NUTM2B-AS1 | hsa-miR-106b-5p | CYCS TGOLN2 |
| PCAT1 |  |  |
| WWC3-AS1 |  |  |
| AC104777.2 |  |  |
| AC133785.1 |  |  |
| LINC01422 |  |  |
| MIR146A |  |  |
| AC023347.1 |  |  |
| DLG1-AS1 |  |  |
| EPB41L4A-AS1 |  |  |
| HCG18 |  |  |
| ITPK1-AS1 |  |  |
| LCMT1-AS2 |  |  |
| LINC01426 |  |  |
| NUTM2B-AS1 |  |  |
| PCAT1 |  |  |
| PHKA2-AS1 |  |  |
| AC104777.2 |  |  |
| LINC01422 |  |  |
| MIR146A |  |  |
| AC006076.1 |  |  |
| AC073130.1 | hsa-miR-125a-3p | PGK1 |
| AC144450.1 |  |  |
| ACTA2-AS1 |  |  |
| C1QTNF1-AS1 |  |  |
| DPYD-AS1 |  |  |
| FAM138D |  |  |
| HCG14 |  |  |
| HCG24 |  |  |
| ITGB2-AS1 |  |  |
| LINC00350 |  |  |
| LINC00862 |  |  |
| LINC01358 |  |  |
| ST3GAL5-AS1 |  |  |
| TMEM212-AS1 |  |  |
| TSPEAR-AS2 |  |  |
| EPB41L4A-AS1 |  |  |
| HCG18 |  |  |
| ITPK1-AS1 |  |  |
| LCMT1-AS2 |  |  |

| NUTM2B-AS1 |  |  |
| --- | --- | --- |
| LINC01422 |  |  |
| AC073130.1 |  |  |
| TSPEAR-AS2 |  |  |
| LINC00582 |  |  |
| SERHL |  |  |
| LINC01426 |  |  |
| NUTM2B-AS1 |  |  |
| AC133785.1 |  |  |
| AC006076.1 | hsa-miR-16-5p | CYCS HSPA5 KDR PDIA6 TGOLN2 |
| AC144450.1 |  |  |
| C1QTNF1-AS1 |  |  |
| TSPEAR-AS2 |  |  |
| LINC00582 |  |  |
| AC023347.1 |  |  |
| EPB41L4A-AS1 |  |  |
| HCG18 |  |  |
| ITPK1-AS1 |  |  |
| LCMT1-AS2 |  |  |
| LINC01426 | hsa-miR-17-5p | CYCS TGOLN2 |
| NUTM2B-AS1 |  |  |
| PCAT1 |  |  |
| WWC3-AS1 |  |  |
| MIR146A |  |  |
| TSPEAR-AS2 |  |  |
| HCG18 |  |  |
| ITPK1-AS1 |  |  |
| LINC01426 |  |  |
| PCAT1 |  |  |
| AC133785.1 |  |  |
| LINC01422 | hsa-miR-181b-5p | PDIA6 |
| MIR146A |  |  |
| ACTA2-AS1 |  |  |
| TSPEAR-AS2 |  |  |
| SERHL |  |  |
| AC004988.1 |  |  |
| ARHGEF38-IT1 |  |  |
| DLG1-AS1 |  |  |
| HCG18 |  |  |
| ITPK1-AS1 |  |  |
| LCMT1-AS2 |  |  |
| AC104777.2 |  |  |
| AC133785.1 |  |  |
| ACTA2-AS1 | hsa-miR-18a-3p | PGK1 |
| FAM138D |  |  |
| LINC00862 |  |  |
| TSPEAR-AS2 |  |  |
| AC004988.1 |  |  |
| ARHGEF38-IT1 |  |  |
| AC010745.3 |  |  |
| FAM138C |  |  |
| DLG1-AS1 |  |  |
| LCMT1-AS2 |  |  |
| MIR146A | hsa-miR-197-3p |  |
| C1QTNF1-AS1 |  |  |
| TSPEAR-AS2 |  | CYCS |
| AC004988.1 |  |  |
| HCG18 |  |  |

hsa-miR-15a-5p PDIA6

hsa-miR-21-3p

| NUTM2B-AS1 |  |  |
| --- | --- | --- |
| FAM138D |  |  |
| FAM138C |  |  |
| HCG18 |  |  |
| LCMT1-AS2 |  |  |
| LINC01426 |  |  |
| PCAT1 |  |  |
| PHKA2-AS1 |  |  |
| AC104777.2 |  |  |
| ACTA2-AS1 |  |  |
| C1QTNF1-AS1 |  |  |
| FAM138D | hsa-miR-324-5p | PGK1 |
| ITGB2-AS1 |  |  |
| TSPEAR-AS2 |  |  |
| LINC00582 |  |  |
| SERHL |  |  |
| ARHGEF38-IT1 |  |  |
| FAM138C |  |  |
| DLG3-AS1 |  |  |
| NEBL-AS1 |  |  |
| AC023347.1 |  |  |
| HCG18 |  |  |
| ITPK1-AS1 |  |  |
| LCMT1-AS2 |  |  |
| LINC01426 |  |  |
| NUTM2B-AS1 |  |  |
| PCAT1 |  |  |
| PHKA2-AS1 |  |  |
| AC104777.2 |  |  |
| AC133785.1 |  |  |
| LINC01422 |  |  |
| MIR146A |  |  |
| AC144450.1 | hsa-miR-3918 | CYCS |
| ACTA2-AS1 |  |  |
| DPYD-AS1 |  |  |
| FAM138D |  |  |
| ITGB2-AS1 |  |  |
| LINC00350 |  |  |
| ST3GAL5-AS1 |  |  |
| TSPEAR-AS2 |  |  |
| SERHL |  |  |
| AC004988.1 |  |  |
| ARHGEF38-IT1 |  |  |
| AC010745.3 |  |  |
| FAM138C |  |  |
| AC017074.2 |  |  |
| EPB41L4A-AS1 | hsa-miR-548x-3p | HSPA5 PGK1 |
| EPB41L4A-AS1 |  |  |
| HCG18 |  |  |
| LCMT1-AS2 |  |  |
| LINC01426 |  |  |
| NUTM2B-AS1 |  |  |
| PCAT1 |  |  |
| AC133785.1 |  |  |
| LINC01422 |  |  |
| MIR146A |  |  |
| AC006076.1 | hsa-miR-615-3p | CKAP4 |
| AC073130.1 |  |  |

| ACTA2-AS1 |  |  |
| --- | --- | --- |
| DPYD-AS1 |  |  |
| ITGB2-AS1 |  |  |
| LINC00862 |  |  |
| ST3GAL5-AS1 |  |  |
| TSPEAR-AS2 |  |  |
| AC004988.1 |  |  |
| FAM138C |  |  |
| DLG1-AS1 |  |  |
| ITPK1-AS1 |  |  |
| PCAT1 |  |  |
| MIR146A | hsa-miR-92a-3p | PPIB |
| FAM138D |  |  |
| ITGB2-AS1 |  |  |
| FAM138C |  |  |
| EPB41L4A-AS1 |  |  |
| HCG18 |  |  |
| ITPK1-AS1 |  |  |
| LCMT1-AS2 |  |  |
| LINC01426 |  |  |
| PCAT1 |  |  |
| PHKA2-AS1 |  |  |
| AC104777.2 |  |  |
| AC133785.1 | hsa-miR-93-3p | CYCS HSPA5 |
| MIR146A |  |  |
| AC073130.1 |  |  |
| ACTA2-AS1 |  |  |
| FAM138D |  |  |
| LINC00350 |  |  |
| TSPEAR-AS2 |  |  |
| SERHL |  |  |
| AC010745.3 |  |  |
| FAM138C |  |  |
| AC023347.1 |  |  |
| EPB41L4A-AS1 |  |  |
| HCG18 |  |  |
| ITPK1-AS1 |  |  |
| LCMT1-AS2 |  |  |
| LINC01426 |  |  |
| NUTM2B-AS1 |  |  |
| PCAT1 |  |  |
| WWC3-AS1 |  |  |
| AC133785.1 | hsa-miR-93-5p | CYCS TGOLN2 |
| LINC01422 |  |  |
| MIR146A |  |  |
| C1QTNF1-AS1 |  |  |
| FAM138D |  |  |
| LINC00350 |  |  |
| TSPEAR-AS2 |  |  |
| SERHL |  |  |
| AC004988.1 |  |  |
| LINC00613 |  |  |
